# Elevation of a patient's trunk and legs does not influence length of stay in the postanesthesia care unit

**DOI:** 10.1590/S1516-31802004000500007

**Published:** 2004-09-01

**Authors:** Otávio Omati, Fábio Ely Martins Benseñor, Joaquim Edson Vieira

**Keywords:** Anesthesia, Supine position, Anesthesia recovery period, Position modalities, General anesthesia, Anestesia, Período de recuperação da anestesia, Modalidades de posição, Decúbito dorsal, Anestesia geral

## Abstract

**CONTEXT:**

Patient recovery time after anesthesia depends on problem-oriented monitoring and individual assessment.

**OBJECTIVE:**

To investigate the influence of patient positioning on post-anesthesia recovery time.

**TYPE OF STUDY:**

Retrospective.

**SETTING:**

Post-anesthesia care unit, Hospital das Clínicas, São Paulo.

**METHODS:**

Data were obtained from patients recovering from anesthesia in a supine horizontal position or with their trunk and legs elevated at 30 degrees. Data were recorded every 30 minutes. The start time was considered to be the admission to the unit, and the final measurement was taken when the patient reached an Aldrete-Kroulik index of 10. The length of time until discharge was recorded.

**RESULTS:**

442 patients recovering after general (n = 274) or regional anesthesia (n = 168) were assigned to be kept in a supine position or with their trunk and legs elevated. There was no difference in the medians for non-parametric results, between supine position (75 min, n = 229) and trunk and legs elevated (70 min, n = 213); p = 0.729. Patients recovered faster from regional anesthesia with trunk and legs elevated (70 min) than in the supine position (84.5 min), although not significantly (p = 0.097). There was no difference between patients recovering from general anesthesia, no matter the positioning (70 min; p = 0.493).

**DISCUSSION:**

Elevated legs may supposedly improve venous return and cardiac output since spinal anesthesia blocks sympathetic system and considering leg-raising has been shown to improve cardiac output from hipovolemia. Our findings did not support this hypothesis. Some limitations included a retrospective collection of data that did not allow randomization for recovery position and the unregistered duration of the exposure to the anesthetic drugs.

**CONCLUSIONS:**

There was no difference in anesthesia recovery time in relation to positioning patients supinely or with trunk and legs elevated.

## RESUMO

**CONTEXTO:**

A recuperação após anestesia depende de monitorização padronizada e de avaliações individuais.

**OBJETIVO:**

Investigar a influência do posicionamento do paciente sobre o tempo de recuperação pós-anestesia geral e regional.

**TIPO DE ESTUDO:**

Retrospectivo.

**LOCAL:**

Unidade de Recuperação Pós-Anestésica, Hospital das Clínicas de São Paulo.

**MÉTODOS:**

Pacientes receberam cuidados de enfermagem sob supervisão de anestesiologista na Unidade de Recuperação Pós-Anestésica quando em posição supina ou com tronco e pernas elevadas em 30 graus a cada 30 minutos. O início da contagem de tempo foi na admissão à unidade e a última medida foi tomada quando paciente recebeu nota 10 na escala de Aldrete-Kroulik. O período de tempo até receber alta foi registrado.

**RESULTADOS:**

442 pacientes recuperaram-se após anestesia geral (n = 274) ou regional (n = 168), posicionados em supino ou com tronco e pernas elevadas. A mediana para resultados não-paramétricos não mostrou diferença no tempo de permanência quando em supino (75 min, n = 229) ou com tronco e pernas elevados (70 min, n = 213, p = 0,729). Pacientes recuperaram mais rapidamente de anestesia regional quando com tronco e pernas elevadas (70 min) do que em supino (84.5 min) embora sem significância estatística (p = 0,097). Não houve diferença na recuperação da anestesia geral em qualquer posição investigada (70 min, p = 0,493).

**DISCUSSÃO:**

A elevação das pernas em posição supina pode aumentar o retorno venoso pois a anestesia do neuro-eixo bloqueia o sistema simpático e essa manobra mostrou-se capaz de promover melhor débito cardíaco durante hipovolemia. Os achados deste estudo não comprovam esta hipótese. No entanto, algumas limitações deste estudo se aplicam, como uma coleta retrospectiva, impedindo aleatorização dos grupos de recuperação, bem como a ausência de registro do tempo de exposição aos anestésicos.

**CONCLUSÕES:**

Não houve diferença para o tempo de recuperação pós anestesia considerando o posicionamento do paciente.

## INTRODUCTION

Patient recovery time after anesthesia depends on problem-oriented monitoring and individual assessment. The post-anesthesia care unit provides state-of-the-art setup, equipment and facilities to care for patients recovering from anesthesia.

The type of anesthesia administered has little influence on the need for post anesthesia care. This implies that the post-anesthesia care unit may sometimes be overloaded, thereby imposing the risk of jamming of the patient flow through this hospital unit. In addition, such overloading in this unit may delay the hospital's operating room schedule. Among the reasons for low bed availability in the post-anesthesia care unit are clinical assessments that may be slow-paced and low discharge rates due to reduced nursing staff availability.^1^

Ideally, all the patients coming from the surgical theater should be indicated for recovery within the post-anesthesia care unit. Patient transportation and admission are under the responsibility of anesthesiologists, who perform a first physical evaluation and give guidance to nursing staff for them to follow clinical recovery patterns, as indicated by the Aldrete-Kroulik modified scale.^2^

Ponhold and Vicenzi have shown that there was lower incidence of bradycardia during recovery from spinal anesthesia when patients were allowed to stay in a position with their trunk and legs elevated at 30 degrees (hammock position).^3^ Since a stable heart rate is one of the determinants of full recovery from anesthesia within the post-anesthesia care unit, it seemed reasonable to consider whether the patient position could influence the duration of the recovery during the post-anesthesia care period.

The distinct supervision care adopted by some anesthesiologists, in relation to the post-anesthesia patient position, has created a natural environment for investigating whether different patient positions would give rise to differences in the time required for recovery. The objective of this study was to investigate whether modifying the patient's position when recovering from anesthesia would shorten the postoperative recovery time in the post-anesthesia care unit.

## METHODS

The present study was performed at Hospital das Clínicas, Universidade de São Paulo, a tertiary-level public hospital. The study protocol was approved by the institution's Ethics Committee. This investigation was designed as a retrospective study, since there would not be any possibility of intervention regarding the recovery position for the patients admitted to the post-anesthesia care unit. Patients admitted to the post-anesthesia care unit had their post-anesthesia recovery time recorded by means of the nurse's registering system in that unit. Only the patients kept in the supine horizontal position or with their trunk and legs elevated at 30 degrees (hammock position) were considered for this study. Patients in any other position were not included. Pregnant women and patients under the age of twelve years old were also excluded. All patients in this unit were positioned on a bed with no pillow.

Patients’ demographic data and the type of anesthesia they had been given were recorded. The recovery period was defined as the period between admission to the post-anesthesia care unit and the time when the discharge criteria according to the Aldrete-Kroulik scale were reached. It did not include the period between reaching the discharge criteria and the moment when the patient left the unit. The nursing staff assessed patients on the AldreteKroulik scale every thirty minutes. Patients received analgesics and intravenous fluid when indicated by the anesthesiologist responsible for the post-anesthesia care unit.

The ideal discharge moment was considered to be when the patient reached a level of 10, according to the modified Aldrete-Kroulik post-anesthesia recovery scale. This scoring system includes the following factors (scores indicated in brackets):

1. Limb movements: no movement in any of the four limbs (0), no movement in two limbs (1), movement restored in all limbs (2);
2. Awareness response: not responding to his/her name (0), a response to his/her name (1), completely awake (2);
3. Blood pressure measurement: less than 20% of what was recorded before anesthetizing (0), between 20% and 50% (1), more than 50% of the pre-anesthesia blood pressure (2);
4. Peripheral hemoglobin saturation: less than 90% with oxygen supply under facial mask (0), more than 90% with oxygen supply under facial mask (1), more than 90% in room air (2);
5. Breathing pattern: apnea (0), hypoventilation or dyspnea (1), deep breathing and cough capability (2).

The sum of the Aldrete-Kroulik criteria can reach a maximum of ten, thereby establishing a patient's final condition as fully recovered from anesthesia.^2^ However, the discharge criteria also include well-established general conditions such as absence of nausea or vomiting, and also good control over pain.

The lengths of stay were compared via the Mann-Whitney rank sum test, since there was no normal distribution for the recovery time, and the significance level was set at 95%. For patients under regional anesthesia, the Kruskal-Wallis analysis of variance (ANOVA) of ranks and Dunn's test were performed. Demographic data was evaluated using Student's t test, with the significance level set at 95%. [when p value is < 0.05]

## RESULTS

During the months of January through March 2002, approximately 1,050 patients were admitted to the post-anesthesia care unit of Hospital das Clínicas. From this total, 442 patients were included in this study protocol, of whom 229 were kept in a supine position and 213 with their trunk and legs elevated.

Of these 442 patients, 274 had undergone general anesthesia and 168 regional anesthesia (with bupivacaine). The patients who received regional anesthesia were significantly older (50.2 ± 16.1) than those under general anesthesia (42.5 ± 17.3), by Student's t test (p < 0.001; mean ± SD). There was no difference in body mass index between patients under regional anesthesia (25.3 ± 5.1) and those under general anesthesia (24.4 ± 4.6) (p = 0.062). In relation to the recovery position utilized, there was no difference for age (p = 0.363) or body mass index (p = 0.310). Age and body mass index data are shown in Table 1.

**Table 1:** Patients’ age and body mass index (BMI) (mean ± standard deviation), compared by Student's t test

	Age (years)	BMI
**General anesthesia (n = 274)**	42.5 ± 17.3	24.4 ± 4.6
**Regional anesthesia (n = 168)**	50.2 ± 16.1	25.3 ± 5.1
**p**	> 0.001	0.062
**Elevated trunk and legs (n = 213)**	46.2 ± 17.1	25.0 ± 4.9
**Supine position (n = 229)**	44.7 ± 17.4	24.5 ± 4.7
**p**	0.363	0.310

*n: number of subjects.*

The data on the length of stay in the post-anesthesia care unit were considered to be non-parametric. Thus, the median stay was utilized for the comparison of the supine position with elevated trunk and legs, together with the range from the 25^th^ to the 75^th^ percentile. The results showed that there was no difference in length of stay between patients in the supine position (median = 75 min; range: 50-108) or with elevated trunk and legs (70 min; range: 45-110), by the Mann-Whitney test (p = 0.729).

Patients recovered faster from regional anesthesia when the trunks and legs were elevated (70 min; range: 46-110) than when they were in the supine position (84.5 min; range: 60-130), although this was not statistically significant (p = 0.097). However, there was no difference in patients’ recovery rate following general anesthesia, no matter which position was utilized. It is interesting to notice that, although the recovery time in the supine horizontal position was faster for general (70 min; range: 45-95) than for regional anesthesia cases (84.5 min; range: 60-130) (p = 0.008), patients recovering from regional anesthesia seemed to recover faster with their trunk and legs elevated (p = 0.009) (Table 2).

**Table 2:** Length of stay in post-anesthesia care unit, as median in minutes with range from 25th percentile to 75th percentile, and number of subjects (n)

	Elevated trunk and legs (n)	Supine position (n)	p	Total
***General***	70 [45–108.7]	70 [45–95]	0.493	70 [45–100]
***anesthetic***	(n = 131)	(n = 143)		(n = 274)
***Regional***	70 [46–110]	84.5 [60–130]	0.097	80 [50-120]
***anesthetic***	(n = 82)	(n = 86)		(n = 168)
**p**	0.935	0.008		0.056
***Total***	70 [45–110]	75 [50–108]	0.729	
	(n = 213)	(n = 229)		
***Epidural***	60 [43-70]	67.5 [50-102]		
***anesthetic***	(n = 25)	(n = 18)	0.009	
***Spinal***	85 [50.5-115]	88 [60-130]		
***anesthetic***	(n = 52)	(n = 77)		

## DISCUSSION

In the present study, the patient's body position during the post-anesthesia care period did not influence the recovery time at the post-anesthesia care unit. The patients who received regional anesthesia were older than those assigned for general anesthesia in this selected sample. This could be considered to be the result of differences in disease incidence. Regional anesthesia is commonly indicated for surgical procedures on the lower abdomen, such as in the treatment of urogenital disease, for which the prevalence may be higher among the elderly. Nevertheless, the younger patients did not show a better recovery time, for either position investigated. There was no difference in the recovery time after general anesthesia when using the supine position or elevating the trunk and legs position (70 minutes in each case), but the recovery was longer following regional anesthesia when the supine position was utilized. This longer recovery time may have resulted from the large proportion of the sample that underwent spinal anesthesia and were assigned to the supine position.

Spinal anesthesia blocks the pre-ganglionic sympathetic system and hence reduces cardiac output. This physiological effect comes from the reduced stroke volume and vein dilation.^4^ It also reduces the release of catecholamines from the adrenal medulla when a blockade of efferent autonomic pathways is achieved.^5^ These possibilities might add to the physiological supposition that elevated legs may improve venous return and cardiac output. It has been reported, however, that neither leg elevation nor the Trendelenburg body position influences the autonomic cardiac control among wakeful and non-anesthetized volunteers.^6^

Passive leg-raising has commonly been used for the initial treatment of hypovolemic shock. However, there are reports that have pointed out that this does not produce any significant auto-transfusion effect, including in patients with coronary artery diseases.^7^ Although the controversy may remain, the leg-raising maneuver may be capable of increasing stroke volume and cardiac output in hypovolemic humans.^8^ In addition, the stroke index of a patient in a seated position does not differ from the supine position when recovering from exercise, when the systemic vascular resistance index decreases.^9^ Since a patient under regional anesthesia can experience significant decrease in total peripheral resistance in association with reduced venous return, it would be reasonable to consider that the position with trunk and legs elevated can add to the recovery of the patient's hemodynamic homeostasis.

It is important to note that the patients in the regional anesthesia group received only one local anesthetic (bupivacaine), administered either spinally or epidurally. This makes the regional anesthesia group comparable. However, interestingly, it can at the same time bring some limitations. The use of 20 mg of 0.5% bupivacaine as a spinal blockade can result in a period of up to 380 minutes for complete resolution of anesthesia, while a greater quantity (150 mg) of the same solution, for epidural blockade, can reach a longer blockade time of up to 460 minutes.^10^ This would cause a patient who received epidural anesthesia to take longer to recover, considering the Aldrete-Kroulik criteria.

When the patients that received regional anesthesia were classified according to whether they underwent a spinal or an epidural procedure, each subgroup was very small. Surprisingly, it was possible to observe that there were patients with a longer recovery time after receiving spinal bupivacaine (Table 2). Notwithstanding these results, patients in the post-anesthesia unit with their trunk and legs elevated recovered faster than those in the supine position, either after spinal or after epidural anesthesia.

There are some limitations relating to the retrospective method adopted. This design could have imposed a bias on the results, since the duration of the exposure to the anesthetic drugs, which would provide better control over the anesthesia technique, was not recorded. To address the possibility that the quantity of anesthetic drugs might influence recovery time, a prospective and randomized study would bring greater quality to further investigation. Also, the retrospective collection of data did not allow randomization to be performed, although the final distributions of patients’ positions were close for the same group considered (regional or general anesthesia). Finally, the time intervals for measuring the Aldrete-Kroulik index (every 30 minutes), might have imposed some degree of imprecision on the results. A shorter interval would allow a more precise determination of the timing of the anesthesia recovery. The fact that the data were retrospective and came from the nursing records of Aldrete-Kroulik indexes may have blunted their precision.

## CONCLUSION

The patient's body position during the post-anesthesia period did not influence the recovery time at the post-anesthesia care unit. There was no difference in the duration of patients’ post-anesthesia recovery in relation to using the supine position or elevating the trunk and legs at 30 degrees. The faster recovery from general anesthesia could be expected, given the Aldrete-Kroulik criteria.
